# Supplementary material for: Are carbon emissions trading and green financial instruments synergistic? -Comprehensive quantitative research based on content analysis
Source: PLoS One. 2024 Mar 7;19(3):e0298601. doi: 10.1371/journal.pone.0298601 (PMC10919601; doi:10.1371/journal.pone.0298601)
Supplement: S1 Data — (ZIP) [file pone.0298601.s001.zip › Data/地级市Z_F工作报告.zip!/╔╜╢1⁄2/╚╒╒╒╩╨/└┤╫╘╣1⁄2╓┌║┼í╛┬φ┐╦╩2╛▌═°í┐_2016.docx]

各位代表：

现在，我代表市人民政府向大会报告工作，请予审议，并请市政协委员提出意见。

一、“十二五”主要成就和“十三五”奋斗目标

“十二五”时期，全市上下深入贯彻党的十八大及十八届三中、四中、五中全会和习近平总书记系列重要讲话精神，抢抓发展机遇，主动应对挑战，全力推动发展，取得了一系列新成就。经济总量迈上新台阶。初步测算，全市GDP由“十一五”末的1025亿元增加到1670亿元，年均增长10.4%。人均GDP达到57980元，是“十一五”末的1.6倍。一般公共预算收入由55.6亿元增加到121.65亿元，五年翻了一番多。实现规模以上工业增加值580亿元，年均增长12%。社会消费品零售总额突破600亿元，年均增长13.6%。累计完成固定资产投资5517亿元、实际到账外资24.8亿美元，年均分别增长18.7%和11.7%。转型升级迈出新步伐。三次产业比例预计由9.8﹕54.8﹕35.4调整为8.7﹕48.5﹕42.8。特色种植业面积突破150万亩。林木覆盖率达到41%。建成了威亚发动机三、四期，派沃泰变速箱一、二期，日钢高档薄板，大H型钢，森博液体包装纸板等一批临港工业大项目。省级以上名牌产品达到123个。金融业增加值占GDP的比重由3.6%提高到6%。互联网经济迅速兴起。新型城镇化加快推进。基础设施实现重大突破。日照机场建成通航。日照港跻身全国沿海港口第八、世界港口第十一大港，货物吞吐量由2.27亿吨增加到3.61亿吨。瓦日铁路、胶新铁路电气化改造、青临高速、疏港高速、613省道和山海路西延工程建成通车。高标准建设了城市规划展览馆、淄博路道路及地下工程。“沭水东调”工程实现应急调水，日照水库向岚山调水等工程建成通水，市区日供水能力比“十一五”末新增15万方。500千伏巨峰等53项输变电工程竣工投运。社会建设全面进步。新改扩建幼儿园488处、中小学466处，整建制、一次性通过“全国义务教育发展基本均衡县”评估验收。圆满承办了“十艺节”有关赛事。沂蒙画派跻身全国十大知名画派。公共体育、卫生计生服务体系更加完善。全国农村社区管理和服务创新实验区通过结项验收。“六五”普法依法治理任务圆满完成，立体化治安防控体系更加健全，食品药品综合监管不断加强，社会保持和谐稳定。实现创建全国双拥模范城“五连冠”。民生持续改善。各级财政用于保障改善民生的投入达529.5亿元，是“十一五”的3.26倍。城镇和农村居民人均可支配收入分别达到26200元和12300元。新增城镇就业24.7万人，转移农村劳动力14.8万人。实现城乡居民基本养老、基本医疗保险全覆盖，9.7万名达到退休年龄的被征地农民享受城镇职工养老保险待遇。新增农村公路通车里程1751公里，行政村自来水普及率达到100%。

这些成就的取得，是省委、省政府和市委坚强领导，市人大、市政协和社会各界监督支持，各级各部门和全市人民团结奋斗的结果。在此，我代表市人民政府，向全市人民，向各级人大代表、政协委员，向各民主党派、工商联、无党派人士、各人民团体，向离退休老领导、老同志，向驻日照人民解放军和武警官兵，表示衷心的感谢并致以崇高的敬意！

各位代表！

从今年起，我国跨入了第十三个五年计划。“十三五”是全面建成小康社会的决胜阶段，也是我市奋力赶超，改变“沿海后发展、欠发达”面貌的关键时期。根据中共日照市委《关于制定日照市国民经济和社会发展第十三个五年规划的建议》，市政府组织编制了《日照市国民经济和社会发展第十三个五年规划纲要（草案）》，提交本次会议审议。

“十三五”时期全市经济社会发展的总体要求是：全面贯彻党的十八大和十八届三中、四中、五中全会精神，以邓小平理论、“三个代表”重要思想、科学发展观为指导，深入贯彻习近平总书记系列重要讲话精神，按照中央“四个全面”的战略布局和省委“一个定位、三个提升”的要求，坚持发展第一要务，坚持创新、协调、绿色、开放、共享的发展理念，坚持稳中求进、转型升级工作主基调，积极适应、把握、引领经济发展新常态，着力突破园区、旅游和县域经济，统筹推进经济建设、政治建设、文化建设、社会建设、生态文明建设，全面提升日照综合实力、区域竞争力、创新能力、发展活力，加快建设海洋特色新兴城市，提前全面建成小康社会。

“十三五”的奋斗目标是：实现“两个提前、三个高于、七个显著提升”。即，地区生产总值、城乡居民人均可支配收入提前比2010年翻一番；地区生产总值、高新技术产业产值、户籍人口城镇化率增幅高于全省平均水平；综合实力、产业竞争力、人民生活水平、社会文明程度、生态建设水平、社会治理体系和治理能力现代化水平、城乡一体化水平显著提升。

为便于各位代表审议，现就有关问题说明如下：

（一）关于增长速度和着力点问题。发展是解决一切问题的基础和关键。没有经济发展作基础，全面建成小康社会就无从谈起。建市以来，我市经济持续快速增长，但从总体来看，仍是一个“沿海后发展、欠发达”城市，GDP、财政收入等指标列全省第16位，人均GDP、人均财政收入低于全省平均水平。日照地处东部沿海，是国家确定的重点开发区域，发展优势多、空间足、潜力大。在发展问题上，我们一定要对“沿海后发展、欠发达”的基本市情保持清醒的认识，始终保持战略定力。“十三五”时期，要坚定不移地把“生态立市、工业强市、旅游富市、开放活市、人才兴市”摆在突出位置，把扩大总量、做大“蛋糕”作为首要任务，努力保持一个较快的发展速度，坚决打好翻身仗！

据测算，今后几年，我市只需保持7%左右的增长速度，到2018年即可实现GDP比2010年翻一番。为体现自我加压、负重奋进，《纲要（草案）》提出，到“十三五”末人均GDP要达到全省平均水平。按此测算，未来五年GDP年均增幅必须达到9%以上。实现这个目标，是奋力赶超，改变日照“沿海后发展、欠发达”面貌的需要，更是全市人民的殷切期盼。只要我们下定决心，苦干实干，就一定能够如期胜利实现！

（二）关于“生态立市”问题。生态优势是日照的战略资源、立市之本。保护好日照的生态环境，是对历史负责，对人民负责，对子孙后代负责，也是打造宜居城市、旅游城市，实现永续发展的基础。随着工业化、城镇化进程的加快，我市生态压力越来越大，人民群众改善生态环境的呼声越来越高。尽管采取了大量措施，环境质量逐年改善,但与全市人民的期盼还有很大差距。“十三五”时期，我们要全面落实“绿色发展”理念，坚持“生态立市”，加快建设天蓝水碧、山青水秀美丽日照，让全市人民在获得更多物质财富的同时，享受到更加优美的生态环境！

（三）关于转型升级问题。经济总量小与结构层次低的矛盾并存，是日照现阶段经济的基本特征。2015年，全市钢铁、水泥、发电、石化等重工业产值占比高达65.4%，装备制造业仅占29.6%；高新技术产业产值占比低于全省平均水平12个百分点。在经济进入新常态、结构性产能过剩矛盾凸显的大背景下，盲目扩大产能的路子已经走不通。不发展是死路一条，不转型同样是一条死路！“十三五”时期，我们要按照供给侧改革的要求，坚持扩总量、调结构两手发力，着力构建以现代农业为基础、先进制造业和现代旅游业为支柱、现代服务业为主导、高新技术和战略性新兴产业为引领的现代产业体系，推动日照经济向中高端迈进！

（四）关于“旅游富市”问题。旅游业是“无烟产业”和永远的“朝阳产业”，是“富民、富市、富财政”的黄金结合点，也是推进供给侧改革的重点领域之一。日照拥有“蓝天、碧海、金沙滩”的生态优势，境内海、山、古、林、泉（温泉）兼备，文化底蕴丰富，发展旅游业的条件得天独厚。随着国内消费升级和我市航空高铁时代的相继到来，我们迎来了旅游业大发展的春天!“十三五”时期，我们要高举“旅游富市”的旗帜，把旅游业提升到与工业同等重要的位置，打响“阳光海岸、美丽日照”旅游品牌，加快打造旅游支柱产业！

（五）关于突破水利、交通瓶颈制约问题。水资源匮乏和交通不畅，是制约日照长远发展的两大瓶颈。从水制约来看，日照水资源总量仅占全省的4.9%，是全省唯一无客水来源的城市，区域性、季节性缺水问题十分突出。连续两年的干旱，给我们敲响了警钟。按现状供水能力测算，到2020年，城市和工业用水年缺口将达6300万方，提高水安全保障能力已迫在眉睫！从交通制约看，虽然空中通道已经打通，但陆路交通仍不便捷。“十三五”时期，我们要大办水利、大干交通，集中实施一批水路重大项目，力争早日冲破瓶颈制约，为长远发展提供有力支撑！

（六）关于县域发展问题。县域经济薄弱，是日照的突出短板。2014年，在全省116个县域单位GDP排名中，岚山排第30位，东港排34位，莒县排55位，五莲排95位；在全省县级财政收入排名中，东港排第25位，岚山排48位，莒县排87位，五莲排96位。县域强，全市强。没有县域的突破，就没有全市的崛起。“十三五”时期，我们要实施新一轮县域经济提升行动，激励县域经济进位争先，到“十三五”末，力争两县主要经济指标达到全省中游水平，两区主要经济指标达到全国百强县水平。

二、2015年的政府工作

刚刚过去的2015年，是日照发展史上极不平凡的一年。我们遭遇了国际市场低迷和国内经济下行的双重挤压，经受了贸易融资风险和持续干旱的双重冲击。困难前所未有，压力前所未有。面对严峻挑战，我们坚持事不避难，主动担当，深入开展“解放思想求创新、转型升级促发展”系列活动，突出抓了七件大事：

（一）把“突破园区、聚力招引”确立为全市的头号工程，掀起新一轮大开放、大招引、大发展的热潮。针对发展氛围不浓，缺乏新的经济增长点，后续增长乏力的问题，我们经过深入调研论证，确立了“突破园区、聚力招引”头号工程，出台了市委、市政府《关于突破园区、聚力招引的实施意见》及“1+5”配套文件，推出了38条“真金白银”的政策；隆重召开具有里程碑意义的千人动员大会，吹响了新一轮大开放、大招引、大发展的进军号，打响了向落后宣战的战役!

市委、市政府这一重大决策部署，在全市引起了强烈反响，使各级干部受到了强烈冲击，增强了坐不住、等不起、慢不得的危机感、责任感、压力感，唤醒了干事创业、求新求变的激情，形成了你追我赶、热火朝天的可喜局面。放眼日照大地，全市上下一条心、一股劲，一心一意抓发展的热潮，正在蓬勃兴起！到去年底，全市共招引落地项目189个，实际到位资金199.43亿元；对上争引无偿资金87.94亿元，比上年增长39.08%；举行海外高层次人才日照行、高层次人才故乡行，引进高层次创新团队40个、创业团队20个、创新人才162人；引进技术专利62个。

（二）推出“五抓”组合拳，打好遏制经济下行阻击战。去年上半年，受多种因素的叠加影响，我市经济增长一度跌到建市26年来的最低谷。为尽快实现止跌回升，我们出重拳、下猛药，打出了“五抓”系列“组合拳”。在抓投资方面，夯实市级领导包保大项目责任制，100个市级大项目完成投资433.9亿元，百项重点工业项目完成投资135亿元，固定资产投资增长14.5%。在抓稳企方面，梳理出两批涉及96家企业的200个事项，下单督办，限期办结。在抓融资方面，设立了2亿元的市级股权投资引导基金，全年实现债券、股票融资135.3亿元，比上年增长40.6%；与省工农中建四大银行签署战略合作协议，四家银行承诺，三年给予我市1200亿元融资支持；与省经开投签署战略合作协议，未来三年向我市投资100亿元以上；大力推进直接融资，3家企业在“新三板”挂牌，窝窝商城在纳斯达克上市。在抓增收方面，深入挖掘税收潜力，强化城镇土地使用税清理和营业税清收。在抓“双创”方面，设立了日照创业大学和大学生创业孵化基地，全年发放创业贷款8.89亿元，扶持创业7030人，带动就业3.3万人。新注册登记市场主体33946户，增长20.14%。

经过持续发力，“五抓”疗效逐渐显现，经济回升速度比预料得要好。预计全年GDP增长7.5%，比一季度回升1.2个百分点。规模以上工业增加值增长8.5%，增幅居全省前列。一般公共预算收入增长9.5%，比一季度回升4.5个百分点，增幅居全省第11位；税收占比79.5%，居全省第4位。本外币贷款余额比年初增长14.4%，增幅居全省第一。房地产投资增长18.4%，增幅居全省第一。集装箱吞吐量增长16.2%，原油吞吐量增长20.6%，增幅居全国沿海主要港口前列。

（三）主动应对贸易融资风险，稳定经济金融秩序。2014年8月，我市贸易融资风险开始暴露，之后逐渐扩散蔓延。涉险企业担保关系复杂，融资额巨大。任何一家处置不当，都将产生连锁反应，引发区域性金融风险，给我市实体经济和金融安全带来沉重打击，让日照的经济发展倒退若干年！这是一场输不起的战役！

在这决定经济命运的紧要关头，我们采取断然措施，召开了具有重要转折意义的“3•17”会议，立下了化解风险的时间表、路线图、军令状，成立了金融发展与稳定领导小组，向各家重点企业派出工作组，建立了对区县的约谈机制、对银行的奖惩机制、对企业逃债跑路的严打机制，成功控制了风险外溢，避免了塌方式爆发，守住了不发生系统性、区域性风险的底线。下半年以来，及时把工作重心转移到重点企业分类处置上，为大地金属项目和晨曦集团石化项目组建2个银团，发放贷款18.6亿元；处置银行不良资产61.4亿元、转化不良资产41.4亿元；立案查处逃废银行债务案件36起，刑事拘留24人，挽回直接经济损失1.73亿元、间接经济损失3.7亿元，使金融生态得到优化，信用环境得到净化。

（四）向落后的体制机制动刀子，深化改革激发活力。改革是祛病根、激活力的利器，改革推进到哪里，哪里就会焕发勃勃生机。一年来，我们坚持问题导向，哪里体制不顺、机制不活，就向哪里动刀子，一大批改革成果落地生效。投融资改革方面，在城投集团快速健康发展的基础上，新组建了水务集团、交通发展集团、土地储备开发集团、公交集团。创新组建了金贸资产管理公司，推出“动产质押融资业务解决方案”，化解了贸易企业融资和银行授信“两难”。城中村改造方面，按照政府垄断土地一级市场，让利于区政府、村居和居民的原则调整政策，启动了新一轮城中村改造，全年共拆迁房屋12576户。公用事业改革方面，大力推进水务一体化改革，打破了“多龙治水”格局，形成了统一高效的水市场秩序。改革市区供热体制，实现了减少环节、降低成本、节能减排的多赢效应。国有企业改革方面，设立了新的国资委，实施了国有企业负责人薪酬制度改革，强化了对国有资产的监管。事业单位分类改革方面，加快了经营性事业单位转企改制步伐。教育改革方面，将市直义务教育学校下放，市区普通高中上收，实行中等职业教育资源整合，设立高校工委，完成了中小学校长职级制改革。综合执法改革方面，下放城市管理、人防、旅游、文化等执法权限，理顺了市区执法体制。价格改革方面，实行了阶梯水价、阶梯气价。按照上级部署，启动了机关事业单位工资和养老保险制度改革、县以下公务员职务与职级并行制度改革。

（五）顺应全市人民热切期盼，打好创城突出问题集中整治攻坚战。针对新老城区“两重天”、违章建筑“乱搭建”、交通秩序“闯抢堵”、农贸市场“脏乱差”、老旧小区“杂乱破”等突出问题，组织开展了声势浩大的创城突出问题集中整治行动，共拆除违章建筑109.33万平方米，整治老旧小区541个，新建市场26个，改造提升市场20个，维修道路114.7万平方米，清运积存垃圾38万吨，取缔占道、店外经营摊点4.28万处，整治规范沿街广告牌5.77万处，施划标线33万平方米，施划停车泊位5.3万个。出台省内首部城市管理办法，建立了防止问题反弹的长效机制。市民普遍反映，路宽敞了、小区变新了、心里不堵了。同步推进乡镇街道驻地环境综合整治和城乡环卫一体化，清理村庄积存垃圾49万吨，新增农村公路绿化面积130万平方米，第一、二轮美丽文明乡村行动片区共覆盖1975个村庄，占全市村庄的70%。坚持“疏堵结合、处保配套”，大力开展市区三轮车、四轮代步车集中整治行动，配套建设公共自行车站点200个，投放公共自行车3000辆，购置新能源公交车50辆，有效整治了三轮车乱象，广大市民纷纷点赞。以不战则已、战则必胜的坚定决心，铁拳整治卧龙山，坚决拔掉违建“钉子”，还市民一个赏心悦目的后花园。

（六）打响林水大会战，加快建设山青水秀美丽日照。为积极响应中央绿色发展号召，改善生态环境，我们隆重召开林水会战动员大会，向全市发出了“苦干三年打好林水大会战，建设山青水秀美丽日照”的动员令，推出了“八大绿化工程”、“六大兴水工程”作战图。这次大会是一次向荒山贫水宣战的誓师会，展示了全市人民重整山河的雄心壮志，在我市林业、水利发展史上具有重要里程碑意义！全市各级积极响应，迅速行动，一幅为荒山披绿、向大自然要水的壮丽画卷，在5359平方公里的土地上全面展开！

在打好林水大会战的同时，全面实施生态企业及片区、生态海洋、生态流域、生态山体、生态走廊、生态乡村“六项生态工程”，加大污染治理力度，全年“蓝天白云，繁星闪烁”天数达到298天，比上年增加15天。成功创建为全国生态保护与建设示范区，成为全国唯一获此殊荣的沿海地级市。

（七）在全市掀起效能风暴，向不担当、不作为、慢作为、乱作为现象开刀。为助推“突破园区、聚力招引”持续升温，我们隆重召开效能建设大会，确立了打造“干部作风最好、办事效率最高、营商环境最优”城市的奋斗目标。大力推进简政放权，编制公布了行政审批、行政权力、政府部门责任“三张清单”，全面取消非行政许可审批事项，向园区下放市级经济管理权限141项，向区县下放53项。大力推进审批事项“串联”改“并联”，企业注册实现“三证合一、一照一码”，启用了新一代基本建设“一表通”系统和投资在线监管平台，市级网上审批大厅上线试运行。出台《政务环境和效能问责办法》，以铁的决心、铁的手腕开展问责，努力让“效能风暴”刮出声威，让机关痼疾无处藏身！全年共立案查处违纪违法案件371件，给予政纪处分389人；查处违反中央八项规定精神问题26起，处理49人；查处不担当、不作为、慢作为、乱作为典型案件21起，问责23人。

在集中力量解难题、抓大事的同时，我们还统筹抓了以下工作。 “三农”工作方面，特色种植业产值占比超过70%，新型农业经营主体发展到7681个，农村土地承包经营权确权登记颁证工作基本完成，193个村完成集体资产股份合作制改革试点。耕地保护面积连续19年保持动态平衡。对外开放方面，成功举办中国—中亚合作论坛，与中亚五国企业合资成立了日照港中亚国际物流园区，海铁联运集装箱比上年增长181.6%。岚桥集团成功收购澳大利亚达尔文港。日照经济技术开发区创新建设了现代创业中心、工业邻里中心、外商服务中心。民生工作方面，新建236个村的自来水工程，解决了37.62万人的饮水安全问题。完成231公里农村公路大修。实施移民扶持项目323处，实现农村脱贫3.3万人。建立了职工长期医疗护理保险、职工和城乡居民大病保险制度，完善了城乡困难居民医疗救助、临时救助办法。为39.42万特殊人群实施了免费查体。完成县乡公路安全生命防护工程882.4公里。社会稳定方面，及时果断处置“7•16”石大科技燃爆事故，保障了人民群众生命安全。违法犯罪警情连年下降，治安局势持续稳定。信访维稳工作取得新成效。精神文明建设方面，精心打造“德润港城、善行日照”和“日照好人”文明道德品牌。市图书馆建成启用。成功举办第五届“市运会”。积极推动军民融合深度发展。妇女儿童、红十字、老年人、残疾人、民族宗教、打击走私、司法、仲裁、国家安全、史志、档案、气象、援疆、扶贫协作黔江区等工作取得新成绩。

各位代表！

过去的一年，我们紧紧依靠全市人民的支持参与，打赢了一场场攻坚克难的硬仗，唱响了改革发展主旋律，鼓舞了士气，激发了斗志，锻炼了队伍，推动了发展。实践告诉我们，只要勇于担当，主动作为，敢想敢干，就没有攻不破的火焰山，就一定能干出一番新业绩，闯出一片新天地！

三、2016年的政府工作

今年是全面建成小康社会决胜阶段的开局之年，也是推进供给侧结构性改革，落实去产能、去库存、去杠杆、降成本、补短板“五大任务”的攻坚之年。我们要按照“坚持问题导向，立足积极主动，分清轻重缓急，加强协调统筹，实现重大突破”的指导原则，推动经济社会更好更快发展，确保“十三五”规划开好局、起好步。

坚持问题导向，就是瞄准短板所在、瓶颈所限、发展所需、群众所盼，聚焦问题精准发力，下决心冲破瓶颈、化解矛盾、补齐短板；立足积极主动，就是坚持自加压力，主动提升标杆，跳起来摘桃子，力求发展更快、速度更高、质量更强；分清轻重缓急，就是抓住主要矛盾和矛盾的主要方面，集中优势兵力打歼灭战，实现重点工作率先突破；加强协调统筹，就是妥善处理经济与社会、发展与生态、市域与县域、改革发展稳定等各方面的重大关系，增强发展的系统性、整体性；实现重大突破，就是以超常的决心和力度，靠新招、硬招、实招强力推进，使重点难点工作取得实质性突破。

按照上述要求，今年的主要预期目标是：生产总值增长9%左右；一般公共预算收入增长10%；固定资产投资增长15%，社会消费品零售总额增长11%，进出口总值增长3%以上；规模以上工业增加值增长10%；城镇和农村居民人均可支配收入分别增长9%和9.5%左右；新增城镇就业2.6万人，登记失业率控制在3.5%以内；农村劳动力转移就业2万人。居民消费价格涨幅控制在2.5%左右；全面完成节能减排降碳约束性目标。

（一）突出抓好“头号工程”，加快扩总量、调结构。把“突破园区、聚力招引”作为长期战略任务，年内组织两次现场观摩，为“三招三引”再添一把火、再加一把油。

加快园区转型升级。推动国家级开发区瞄准国内外一流园区，实现转型发展；省级开发区依托本地比较优势，打造特色优势主导产业，向集群化发展。今年2月前完成园区“二次规划”，明确7处省级以上园区、日照港区和航空产业园的功能定位，实现“一张棋盘布棋子、一张蓝图全覆盖”。加大项目统筹力度，鼓励发展“飞地经济”，促进园区之间合理分工、错位发展。加强园区投融资平台建设，加大基础设施投入。推进管理、开发主体分离，组建或引进综合发展运营商，提高园区运营效率。减少园区审批环节和执法层级，3月前在日照经济技术开发区设立行政审批局，实行“一枚印章管审批”；6月前在省级以上园区建立跨部门、跨行业的行政执法体制，实行“一支队伍管执法”。深化园区人事分配制度改革，2月前实现日照经济技术开发区、日照高新技术开发区职员制和职员薪酬制改革入轨运行，并稳步在其他省级以上园区推开。

提高招商引资成效。16个产业招商组分别制定“建链、强链、补链”行动方案，建立目标客户库、项目数据库、合作载体库、产业招商地图“三库一图”，瞄准世界500强、中国500强、民营500强和行业10强项目聚力招引。积极培养引进熟悉产业经济、掌握投资政策、通晓商务惯例、精通项目谈判的专业招商人才，建设高素质、复合型、专业化招商队伍。发挥领导招商的表率作用，坚持市级领导带头，主动走向招引第一线。发挥企业的招商主体作用，鼓励企业拿出优质资产、优势产品、潜力项目吸引战略投资者。强化部门招商职责，所有部门都是“第二招商局”，各涉外部门是招引外资的主要部门。年内确保引进投资过5000万元的项目150个以上，过10亿元的10个以上，实际到位资金260亿元以上，实际到账外资5.7亿美元，各区县、园区招商引资实际到位资金比上年增长50%以上。

推进招才引智和招科引技。加大对引才引智政策的宣传落实力度，用好人才引进专项扶持资金，及时兑现高层次人才创业启动资金、科研经费和住房、生活等补贴，解决高层次人才身份编制、职称评聘、子女就学、社会保障等实际困难，2月前人才创新发展院和企业发展研究院启动运行。年内确保引进高层次创业人才或团队20个以上、高层次创新团队40个以上、高层次创新人才400名以上；引进转化国内外先进技术成果80项以上，参与或合作共建博士后科研工作站、国家和省级科技研发服务平台20个以上。

（二）坚持“五抓”不动摇，力促经济运行提速提效。按照“三去一降一补”新要求，找准供给侧改革结合点，不断推出“五抓”新招、硬招、实招，持续放大“药效”。

抓投资重抓龙头项目。围绕14个重点领域，筛选确定一批投资过5000万元的项目，落实市级领导和区县、部门包保责任，确保年内完成投资500亿元以上。（1）大力实施工业转型升级和“互联网+制造业”行动计划，建设投资280亿元的100项重点工业项目。重点抓好山钢日照精钢、日钢高档薄板、宝华新材料、豪迈海工装备、新格变速箱、兴业汽配二期等46个项目，山钢项目年内基本完成土建，开始主体设备安装，明年上半年一期工程一步建成投产。加快日钢产品升级，淘汰小型炼铁高炉，推进第四、第五条ESP生产线和极薄板生产线建设。建成国家碳素结构钢质检中心。优化提升岚山石化产业园、莒县海右工业园。（2）城市建设。年内完成新一轮城市总体规划、商业网点规划、停车场布局专项规划修编，编制海绵城市、给排水、公交规划和高新区等片区控制性详细规划；启动迎宾路快捷路改造和城市公交换乘枢纽、首末站建设，推进医院、中小学等区域公共停车场建设；完成第二套供热管网建设，实施204国道市区段改线、合村路建设等33条城市道路工程，同步配套海滨五路等综合管廊；实施老城区提升工程，打通一批“断头路”和“卡脖子路”；实施海绵城市建设工程；启动汽车西站建设，购置100台新能源公交车，优化公交线网，新建公共自行车站点50个、投放公共自行车3000辆。成立城市综合管理委员会，建设数字化城市管理系统，巩固提升集中整治成果。（3）交通建设。加快推进青日连铁路、潍日高速、岚罗高速、机场高速等在建工程，开工建设奎山综合交通枢纽、鲁南快速铁路日照段、巨峰至岚山港铁路等工程；年内建成30万吨兼顾40万吨级矿石码头和西区四期5个通用泊位，新建续建第3座30万吨级原油码头、山钢项目矿石码头等十大港口重点工程。（4）水利建设。建成“沭水东调”、日照水库增容、长城岭水库并网调水、傅疃河及两城河橡胶坝工程，加快推进“潍水入沭”、海水淡化、森博浆纸中水回用前期工作。（5）电力建设。抓好220千伏傅疃等22项输变电工程和岚山、莒县、五莲4个光伏发电、2个生物质发电项目。

抓稳企重抓降本增效。开展降低实体经济企业成本行动，减少企业制度性交易成本、税费负担、社会保险费及煤电、物流等成本。积极推行随机抽查监管，解决检查任性和执法扰民、执法不公、执法不严等问题。引导企业向先进企业看齐，深化内部人事分配制度改革，推行精细化管理，减少跑冒滴漏。继续开展“点对点、面对面”涉企问题排查，进一步解决好去年结转的问题。对资不抵债、难以救活的“僵尸企业”和去产能企业，坚持多兼并重组、少破产清算，做好职工安置工作，减少社会震荡。

抓融资重抓直接融资。推动沪鸽口腔、海恩锯业、领信科技等企业到“创业板”或“中小板”上市，推进日照港主业整体上市，年内完成股改企业60家，实现“新三板”挂牌5家。通过企业债、公司债、中期票据、短期融资券等工具，加大债券融资力度，年内力争债券、股票融资150亿元以上。强化政银企合作，用好财政贴息、财政奖补、过桥贷款、风险补偿等手段，引导银行业金融机构加大对企业信贷支持力度，全年社会融资规模不低于300亿元。积极引进市外金融机构，推动民生、光大、恒丰、平安、临商银行年内开业运营。扎实推进贸易融资风险化解，加快重点企业分类处置，加大不良资产打包转让核销力度，进一步优化金融生态环境。

抓增收重抓开源挖潜。加强对电子商务等新商业模式的税收征管，加大城镇土地使用税专项清理和营业税清收力度。支持企业实行主辅分离，将服务业从主业中剥离，形成独立纳税主体。搞好税式支出管理平台、综合涉税信息管理平台建设，完善国税地税联合办税机制，堵塞征管漏洞。加强财税队伍建设，提高税收征管水平。积极争取政府存量债务置换额度，防范债务风险。

抓“双创”重抓扶持服务。抓好市级公共创业孵化基地、青年创业社区、日照经济技术开发区现代创业中心、莒县高层次人才创新创业园等“双创”平台建设，完善“五补一贴一奖”政策，支持高校毕业生、专业技术人员、高层次人才、农民工等重点群体创新创业。年内发放创业担保贷款6亿元，完成创业培训5000人，新增创业实体2.5万户，带动就业6万人。

（三）加快培育旅游支柱产业。以滨海资源开发、山岳景区提升、乡村旅游升级为重点，加快构建全域发展的大旅游格局。

坚持规划引领。编制全域旅游总体规划，明确旅游业发展的总体布局和重点任务，以旅游的理念对市域进行全景化打造，建设宜居、宜业、宜游城市。编制东方太阳城、太公岛保护性创意开发、海滨森林公园改造提升等重点项目规划，引领打造精品旅游项目。

强化精品项目带动。由城投集团对奥林匹克水上公园进行“二次开发”，建成激光秀、尚城旅游小镇一期项目，建设第四代海洋馆和四季花鸟园。整合提升梦幻海滩、万平口、灯塔广场等区域，打造海滨旅游高端景区。改造提升五莲山、浮来山、河山、龙门崮等山岳旅游项目，建设海洋牧场、渔人码头、渔乐湾等滨海旅游项目，开工国际会展文化产业园、旅游装备展销基地、黄海影视基地、温泉小镇、石老山养生度假庄园、白鹭湾大地艺术村等新业态旅游项目。开展“农家乐”星级评定行动，推动乡村旅游升级改造。

打造国内领先的旅游环境。实施智慧旅游城市、旅游集散中心、旅游道路通达和旅游服务标识、旅游休闲街区、度假酒店集群、旅游厕所等工程，重点抓好4处游客集散中心、3处特色街区、3家高端度假酒店建设，新建旅游厕所310座、改建2103座；建立与国际通行规则相衔接的旅游服务标准体系。

理顺体制机制。成立旅游发展委员会，加强对旅游业发展的总体谋划和综合协调。整合组建、鼓励发展国有、民营文化旅游产业公司。设立旅游发展引导资金。推进国有景区所有权、管理权、经营权“三权分离”，激发景区发展活力。加大营销力度，制定整体推广方案，策划推出精品旅游线路，大力拓展中高端客源市场。

（四）用好改革这把“撒手锏”，革除障碍增添活力。把发展的难点作为改革的重点，全力破解体制机制障碍。（1）投融资改革。用好日照港集团、城投集团、水务集团、交通发展集团、公交集团、土地储备开发集团、机场建设投资公司等事业发展和融资平台，年内融资到位100亿元以上。设立PPP项目储备库和实施库，扩大政府与社会资本合作。积极推进新型农村合作金融试点，加快建设专业化中小企业投融资和众筹服务平台，着力破解“三农”和中小微企业融资难、融资贵。（2）国企改革。出台深化市属国有企业改革、完善国有资产监管体制、规范国有企业负责人履职待遇等系列配套文件。推进国有企业用人制度改革，实行公开招聘、竞争上岗等制度，形成“能上能下、能进能出”的机制；加强日照港财务公司运营管理，开展国有资本投资运营公司试点。（3）行政审批改革。加快转变政府职能，持续推进权力瘦身，每半年组织一轮行政权力精简下放，成熟一批、下放一批；加快审批事项“串改并”，确保办理时限不超过最高法定时限的50%；上半年建成市、县两级网上政务服务平台。（4）财税改革。全面公开政府预决算、部门预决算及“三公”经费预决算。充分发挥市级股权投资引导基金作用，有效吸引社会资本投资。（5）农村产权制度改革。在两县开展土地经营权抵押、担保试点。完成200个村的集体资产股份合作制改革。（6）综合执法改革。按照成熟一项、划转一项和能放则放的原则，扩大综合执法范围，向区县、乡镇下移执法重心。（7）医药卫生体制改革。完善公立医院法人治理结构，建立适应行业特点的人事薪酬制度。在五莲县开展分级诊疗试点。（8）公共资源交易改革。整合扩展现有交易平台，推进公共资源交易法制化、规范化、透明化。（9）住房制度和公车改革。坚持开正门、关后门、堵旁门，推进公务人员住房分配货币化，建立临时周转房和公租房制度。完成党政机关公务用车改革。

（五）构建对外开放新格局。有效做好“一带一路”战略与项目、平台的对接工作，努力构建“双港带动、多园支撑、三外协调”的对外开放新格局。

实施海空双港带动。海港方面，在巩固既有大宗散货运输优势的基础上，实行箱、油、商、工、金“五子登科”，推动港口转型升级，提升核心竞争力，在“南北夹击”的激烈竞争中突出重围！加快日照、岚山口岸整合，打造国际枢纽口岸。按照铁路“南进南出”、货种“北集南散”的目标，加快海港布局调整。推进港区（日照港和开发区）联动、港城产融合，努力把海港打造成重要的经济增长点和税源地。空港方面，坚持“支线运输、通用航空、临空产业”三位一体，加快空港园区建设，积极引进飞机制造、拆解、维修、保养等项目，争取空港口岸开放，开通跨境航线，努力把机场打造成高效便捷的交通枢纽和高端装备制造、现代物流、综合旅游等业态融合崛起的产业龙头，实现点上点金、面上收银。

强化多园支撑。依托中韩汽车零部件产业园，推进中韩产业园招商引资和规划建设。争取成功申建日照综合保税区。抢抓中澳自贸区建设机遇，规划建设国家层面的中澳产业园，大力发展澳大利亚活牛羊、肉制品、奶制品、水产品冷链物流以及木材、矿石进口等业务。推进亚欧、中亚、中蒙通道日照出海口规划建设。

促进三外协调。利用外资方面，重点抓好与国内外行业领军企业的合作，推动与华信、金鹰、豪迈等世界500强、中国500强合作的6个在谈项目尽快落地。用好中韩自贸区建设机遇，积极引进汽车零部件、综合物流等项目。加快建设与国际接轨的法治环境、政策环境、生活环境。对外贸易方面，坚持“优进优出”，推动进出口实现恢复性增长，稳固外贸大市地位。重点抓好4处特色出口基地建设，巩固钢铁、发动机、变速箱等传统优势产品，扩大农用机械、小五金、机电产品出口规模。大力发展跨境电子商务，深入推进与阿里巴巴的战略合作，抓好“一拍档”外贸出口服务平台和日钢等3家企业海外仓建设。全面实施“大通关”服务，建设“单一窗口”电子口岸，促进贸易投资便利化。外经方面，支持企业积极稳妥地走出去，推进产能合作，打造本土跨国企业。鼓励建筑企业开拓“一带一路”沿线市场。

（六）聚力突破县域经济。优化区县发展定位，鼓励区县因地制宜，打好“突破园区、聚力招引”攻坚战，加快培育各具特色的“板块经济”。支持东港区发展高新技术产业和现代服务业；支持岚山区加快钢铁、石化等产业转型升级；支持莒县做大做强特色农业、农产品精深加工、机械制造、石油化工、现代物流等产业；支持五莲县提升发展生态旅游、汽车及现代农用装备、高端制造等产业。加大考核力度，调动区县自我发展的积极性。出台支持县域经济发展的综合性意见，从资金、项目、基础设施、事权划分等方面，强化对县域经济的扶持。推动临港产业新增项目、特色产业项目和“退城进园”项目向区县转移，推动基础设施和公共服务向区县延伸，年内完成潮石路拓宽改造、南沿海路改道、青赵线改建、222省道东港和岚山段改建任务，启动实施206国道莒县和五莲段、222省道东港和五莲段升级改造，争取启动613省道西延二期工程。

夯实“三农”基础。按照“人无我有、人有我优、人优我强、步步领先”的思路，深入实施农业十大产业振兴规划和五个特色产业发展规划，抓好“海洋牧场”、“海上粮仓”建设，加快发展果茶菜、肉蛋奶等高效特色农业。实施藏粮于地、藏粮于技战略，加大高标准农田和高产创建力度，稳定粮食生产，保障粮食安全。大力培育新型农业经营主体和服务主体，支持农户开展土地托管服务，鼓励农民以承包地经营权入股组建土地股份合作组织，年内土地经营规模化率提高到35%。扩大“互联网＋”在农业农村的应用，培育一批特色“淘宝村”。

加快新型城镇化进程。坚持以“人的城镇化”为核心，有序推进外来务工人员市民化、城中村和城边村原有居民市民化、其他农村地区就地转移就业人口市民化，年内户籍人口城镇化率力争提高2个百分点以上。按照“三年攻关、五年收官”的要求，年内抓好市区及岚山、两县76个城中村改造，拆迁房屋26761户，建设棚改安置房26897套。调整城中村居民安置方式，实行划片集中建房安置与货币化补助分散安置相结合，政府不再直接投资建设公租房，改由招标购买办法解决，以此推进房地产去库存。深化户籍制度改革，细化住房、就业、社保、教育、医疗等配套政策，让进城人口享受到完全的市民待遇。积极开展示范镇提升行动，选择夏庄、涛雒、招贤、街头等重点小城镇，加快打造区域次中心。扩大住房有效需求，保持房地产业稳定健康发展。深入开展美丽文明乡村行动，年内建设10个绿化示范镇和100个市级森林村庄，改造危房1500户、旱厕13万户，完成251个村的饮水提质改造，完成农村公路大修200公里，实施农村公路安全生命防护工程600公里，实现农村“三年大变样”。

（七）打好林水大会战。围绕实施“八大绿化工程”，抓好日照水库等13座水库环库林带建设、鲍疃河等13条河流林带新建和完善、山海西路等10条道路绿化提升和2.5万亩荒山绿化，新建两城河口国家级湿地公园和和莒县袁公河、北溪省级湿地公园，发展蓝莓、核桃、大樱桃、黄桃、银杏等特色经济林5万亩，全年新增造林面积15万亩，林木覆盖率提高1个百分点以上。围绕实施“六大兴水工程”，建成4个农田水利项目县工程、2个水土保持项目县工程；实施青峰岭、户部岭水库移民避险解困工程；完成户部岭水库除险加固、窝疃村山洪沟治理，尽快启动沭河莒县段综合治理、青峰岭水库溢洪闸除险加固等项目，开工建设绣针河、巨峰河等中小河流治理工程，推进秀水河、金银河等乡村河道水系生态治理。对林水会战实行挂图作战、挂图督战，确保尽快见到实效。

全面推广“河长制”，实施崮河流域“治、用、保”八项工程，重点抓好香店河、营子河、后楼河等市区河流综合整治，全面消除黑臭水体，实现崮河流域水质稳定达标。继续推进日照港、日钢周边片区环境综合整治，加强重点行业废气、机动车尾气排放和建筑工地扬尘治理，抓好城区裸露土地绿化，力争全年PM2.5浓度下降6%以上。建设改造6处污水处理设施。加强山体、岸线、海洋资源保护，完成卧龙山区域综合整治。严格执行投资项目用地、节能、节水、环保、安全等准入标准，推进闲置低效用地分类处置，实施“腾笼换鸟”和“二次开发”1500亩以上。坚持以水定城，以水定产，以水定需，因水制宜。完成既有居住建筑节能改造65万平方米，新建绿色建筑20万平方米。按照“重而特、重而优、重而安全、重而生态环保、重而附加值高”的思路，淘汰落后产能，推动钢铁、化工等产业转型升级。对化工园区内企业周边不符合安全间距要求的村居实施搬迁改造。严格执行建设项目环评审批负面清单，清单之内的30类项目一律不得引进，坚决不要黑色、带血的GDP！

（八）推动社会民生全面进步。尽心尽力为群众解难题、办实事，让老百姓的生活每年都有新变化，日子一年更比一年好。“两会”闭幕后，我们将把报告提出的民生事项全部列出清单，明确责任单位和完成时限，确保按期兑现，决不失信于民！

把脱贫攻坚作为政府头等大事和第一民生工程，按照“五个一批”的总体要求，积极推进“十大精准扶贫行动”，坚决打好脱贫攻坚战，年内完成农村脱贫2.5万人以上。着眼于打通交通瓶颈，发展高效生态农业和乡村旅游，对莒北、莲西等山区库区研究采取针对性、可行性强的扶贫措施，紧紧拉着老区人民的手，不让一个贫困群众在全面建成小康社会的进程中掉队！

实施农村中小学“全面改薄”和解决城镇普通中小学“大班额”工程，年内投资13亿元，新建学校13所，改扩建学校62所，招聘教师1050名。深化基础教育综合改革，完善中小学校长职级制改革，推动建立现代学校制度、中小学教师县管校聘、教育评价机制等改革。推动日照职业技术学院创建国家优质高职院校，提升山东外国语职业学院、日照海事学院办学层次，加快构建现代职业教育体系。加快实施智慧校园工程。

全面建立困难残疾人生活补贴和重度残疾人护理补贴制度，建成市残疾人康复中心。加大住房保障力度，招标购买公租房300套，投放住房公积金27亿元。深入实施农民工职业技能提升、权益保障、公共服务3年三项行动计划，年内完成就业技能培训2.3万人，帮扶困难群体就业1000人。落实全民参保计划，降低社会保险费率。推进机关事业单位养老保险制度改革。

健全完善城乡医疗卫生服务体系，提高人均基本公共卫生服务经费。积极推动医养结合服务综合体建设。加快推进中医药事业发展。坚持计划生育基本国策，积极应对全面“两孩”政策实施的新情况。建设室外全民健身工程200处以上，举办丰富多彩的全民健身活动。

深入推进平安日照建设，完善立体化治安防控体系，不断提升群众的安全感和满意度。全面落实“七五”普法规划，完善公共法律服务体系。扎实做好新形势下的信访工作，创新社会综合治理。强化食品药品监管体系建设。牢固树立安全生产“底线思维”和“红线意识”，严格落实“党政同责、一岗双责、齐抓共管”和“三个必须”要求，确保安全生产形势持续好转。

坚持文化惠民、文明育心，积极培育和践行社会主义核心价值观，深入开展精神文明系列创建活动，争创全国文明城市。加快构建现代公共文化服务体系，实施“千百重点文化工程”、基层综合性文化服务中心等项目。推进全民阅读。加强文化遗产保护。抓好海洋科普馆建设。加快“钢琴＋”综合体、文化创意产业园、星河科技园等文化产业项目建设。深化双拥工作，落实优抚安置政策，促进军民融合深度发展。做好第三次全国农业普查工作。统筹推进妇女儿童、红十字、老年人、民族宗教、打击走私、司法、仲裁、国家安全、史志、档案、气象、援疆、扶贫协作黔江区等各项工作。

四、打造担当、务实、高效、廉洁政府

各位代表！

过去一年，我们积极应对挑战，主动破解难题，一大批干部在“火线”经受了锻炼和考验，提高了胆识和能力。我们切身感受到，事在人为。加快日照发展，要害在干，关键在人！未来五年，我们要按照“三严三实”的要求，持续推进各级政府作风建设，努力锻造一支对党忠诚、勇于担当、充满激情、能打胜仗的政府干部队伍，切实增强各级政府领导干部的决断力、凝聚力、号召力和工作人员的理解力、执行力、创新力，为“十三五”发展提供可靠保障！

大力弘扬担当精神。去年的实践证明，有些事情看起来很棘手，但只要下决心就能干成，而且越干越敢干、越干越会干、越干越想干！我们要大力培养政府干部的担当精神，激励他们事不避难，在困难面前勇往直前，做敢打敢拼的冲锋陷阵者；直面问题不回避，做无所畏惧的亮剑者；勇于冲破思想牢笼和利益樊篱，做创新求变的改革者；坚持“五加二”、“白加黑”，做忘我工作的无私奉献者。各级政府要健全完善容错机制、保护机制，为担当者撑腰壮胆、遮风挡雨。允许有失误，不允许不干事！重视从突破园区、“三招三引”、项目建设、征地拆迁、城中村改造、改革、维稳等急难险重工作中培养、锻炼、发现干部，让想干、敢干、会干的干部有位子、得实惠，让不担当、不作为的干部没市场！

打造认真、专业、务实的干部队伍。把思路和招数结合起来，对每项工作都精心设计实施路径，拿出管用的招数，切实提高工作成效。严格落实责任制，加大督查力度，确保各项决策落地生根。坚持集体学习与个人自学相结合，提高各级政府领导干部的科学化、专业化、法治化水平，争做专家型领导。利用党校、行政学院的资源，对政府系统中层干部和一般干部实行轮训。坚持谋事要实、创业要实、做人要实，多做打基础、利长远、惠民生的事，创造经得起历史和人民检验的业绩。严格执行《政务环境和效能问责办法》，加大明察暗访力度，对不担当、不作为、慢作为、乱作为的人和事，发现一起，查处一起，决不姑息！

加快建设法治政府。各级政府领导干部要带头尊法、学法、守法、用法，当好法治建设的领头雁，练就依法办事的硬功夫，守住党纪国法的警戒线。认真落实人大及其常委会决议决定，自觉接受人大法律监督和政协民主监督，及时听取各民主党派、工商联、无党派人士、各人民团体和人民群众的意见。把公众参与、专家论证、风险评估、合法性审查、集体讨论决定作为重大决策的必经程序，确保制度科学、程序正当、过程公开、责任明确，最大限度避免决策失误。全面推进政务公开，对土地征用、房屋拆迁、招生就业、人员录用、经费开支等涉及群众切身利益和社会关心的事项，依法向社会公开，让权力在阳光下运行！

坚持守纪律、讲规矩。自觉向党中央看齐，向党的理论和路线方针政策看齐，保持坚定正确的政治方向，做政治上的明白人，在思想上、政治上、行动上，坚定不移地同以习近平同志为总书记的党中央保持高度一致。认真学习贯彻《中国共产党廉洁自律准则》和《中国共产党纪律处分条例》，严格落实党风廉政建设主体责任，管好班子、带好队伍。严格遵守中央八项规定，从严控制公用经费支出，确保财政供养人员和“三公”经费只减不增。主动接受司法监督、行政监察、审计监督、新闻舆论监督和人民群众监督，确保行政权力运行到哪里，监督就延伸跟进到哪里。坚持无禁区、全覆盖、零容忍，保持惩治腐败的高压态势，让想搞腐败的人了断念头，让搞了腐败的人付出惨痛代价！

各位代表！

“十三五”的宏伟蓝图已经绘就，我们站在了新的起点上。让我们紧密团结在以习近平同志为总书记的党中央周围，高举中国特色社会主义伟大旗帜，在中共日照市委的坚强领导下，同心同德，励精图治，努力夺取全面建成小康社会的胜利，开创日照更加美好的未来！
